# Cancer related maternal mortality and delay in diagnosis and treatment: a case series on 26 cases

**DOI:** 10.1186/s12884-017-1639-3

**Published:** 2018-01-05

**Authors:** Jorine de Haan, Christianne A. R. Lok, Joke S. Schutte, Lia van Zuylen, Christianne J. M. de Groot

**Affiliations:** 1 Department of Obstetrics and Gynaecology, VU University Medical Center, De Boelelaan 1118, 1081 HZ Amsterdam, The Netherlands; 2 Department of Oncology, KU Leuven, Herestraat 49, 3000 Leuven, Belgium; 3 Department of Gynaecologic Oncology, Center for Gynaecologic Oncology Amsterdam, Amsterdam, The Netherlands; 4 Department of Obstetrics and Gynaecology, Isala Clinics, Zwolle, the Netherlands; 5 Department of Medical Oncology, Erasmus MC Cancer Institute, Rotterdam, the Netherlands

**Keywords:** Cancer, Pregnancy, Delay, Preterm delivery, Mortality

## Abstract

**Background:**

Cancer during pregnancy is relatively rare but may lead to maternal mortality. We aimed to assess the incidence of cancer related maternal mortality and the neonatal outcome in these patients. Also, doctor- and patient-related delay in cancer diagnosis and therapy among patients with cancer related maternal mortality is assessed.

**Methods:**

Maternal mortality was defined as death during pregnancy or within 1 year after delivery. Data of the Dutch Maternal Mortality Committee was used to calculate the cancer related maternal mortality rate and to assess neonatal outcome in the Netherlands. Delay was scored by ten medical specialist based on case descriptions.

**Results:**

Cancer related maternal mortality rate was 1.23 per 100,000 live births. Delay in either diagnosis or treatment occurred in 65%. Delay in diagnosis was more frequent then delay in treatment, and was mainly caused by health care providers. Only 77% of pregnancies were ongoing, and 65% ended preterm of which 85% was induced.

**Conclusions:**

Avoiding delay in diagnosis and therapy in case of pregnancy related cancer could potentially improve maternal and neonatal outcome. It is therefore essential to increase awareness among health care providers about the occurrence and recurrence of cancer in pregnancy and the possibilities of diagnostic and therapeutic interventions in these women.

## Background

In western countries, maternal mortality, defined as death occurring during pregnancy or within the first year after delivery, is a relatively small but still serious problem. Most women die of pregnancy related problems, but approximately 25% of the maternal deaths are non- pregnancy related. Pregnancies complicated by cancer are a potential threat for both maternal and fetal wellbeing. Incidence of cancer during pregnancy has been estimated to be one in 1000 pregnancies, but due to increasing maternal age and the increasing incidence of risk factors for cancer, e.g. obesity, this incidence is rising [1, 2]. Schutte et al. reported no cancer related maternal deaths between 1983 and 1992 and three between 1993 and 2005 [3].

If standard therapy is started without delay, prognosis of pregnant patients is comparable to non-pregnant patients when corrected for maternal age and stage. Cancer related symptoms can mimic those of physiological pregnancy changes [4]. They may be interpreted by both patient and health care providers as pregnancy related, delaying an accurate diagnosis [5, 6]. This delay can lead to a more advanced stage of disease, causing a higher mortality rate [7].

In the management of pregnant patients with cancer, the unborn child is an important second patient that needs to be taken care of. Treatment regimens must be carefully evaluated in order to ensure fetal safety. Recent studies have shown that several cancer treatments seem to be feasible during pregnancy without increasing the change of congenital malformations [8, 9]. Chemo- and radiotherapy during pregnancy does not seem to affect the neuropsychological development up to 3 years. Unfortunately, substandard and/or delayed treatment and iatrogenic preterm birth is still a major problem in this specific patient group [10]. In fact, preterm delivery is a high risk factor for developmental problems later in life [8].

In advanced stages of disease, treatment is often more extensive and maternal condition deteriorated. Minimizing delay in diagnosis will not prevent all maternal deaths, but the outcome for both mother and neonate is likely to be improved. However, literature on current cancer related maternal mortality is scarce. Therefore, in the present study the incidence of cancer related maternal mortality in the Netherlands will be calculated and the occurrence of doctor- and patient-related delay in diagnosis and therapy will be evaluated. Finally, the neonatal outcome of the children of the mothers that died of cancer during pregnancy will be analysed.

## Methods

This study is a nationwide observational cohort study using the non-public database of the Dutch Maternal Mortality Committee (MMC) after written permission of the board of the committee. The methods of the MMC and definitions of maternal mortality used have been described previously [3]. Briefly, the MMC discusses reported cases, collects missing data and evaluates the avoidability of maternal mortality. Maternal mortality is defined as death occurring during pregnancy or within the first year after delivery. Their database is crosschecked with the Central Bureau of Statistics of the Netherlands (CBS). Missing cases are anonymously added to the database.

### Selection criteria and data collection

Patients registered between 2001 and 2012 by the MMC who died during pregnancy or within 1 year postpartum were screened. We selected the patients that died because of (recurrent) cancer. Patients with cancer related symptoms during pregnancy but diagnosed after delivery were included. If patients died from cancer but only showed symptoms after delivery, they were not eligible. Missing data in the reports of the MMC were completed by review of the medical files. Ten medical specialists from different disciplines, including medical oncologists, gynaecological oncologists and obstetricians, were provided detailed case descriptions, with information on diagnosis and therapy. They scored anonymously whether delay had occurred and whether this was doctor- and/or patient-related delay. Delay was defined as an extension of the period between symptoms/presentation and diagnosis, and diagnosis and treatment, compared to optimal care based on expert opinions. For the patients with recurrent disease during pregnancy, delay was assessed from the moment of the new onset of symptoms caused by recurrent disease. Delay due to other primary health care providers like midwifes were considered to be doctor-related delay.

### Statistical analysis

Because of the observational nature of this study, statistical analysis was restricted to descriptive statistics and the evaluation of inter-observer agreement with a kappa score. Because our data was scored by a fixed number of observers using a numeric scorings system the Fleiss kappa score was used.

## Results

### Incidence and maternal mortality rate

Between 2001 and 2012, 32 cancer related maternal deaths were reported to the MMC. Four of these cases were excluded: two patients were treated with chemotherapeutic agents for other indications than malignant disease, one patient had a benign brain tumour and one patient became symptomatic after delivery. Two additional anonymous cases were reported by the CBS, which were included but could only be used to calculate the incidence of cancer related maternal mortality. The 26 remaining cases were available for analysis of delay. With an average number of 188,604 live births per year (range 202,603 in 2001 to 175,959 in 2012) in the Netherlands, the Dutch cancer related MMR between 2001 and 2012 was 1.23 per 100,000 live births [11].

### Patient characteristics

Brain tumours, gastro-intestinal tumours and melanomas were the most common types of cancer causing maternal mortality. Patient characteristics are shown in Table 1. The median maternal age at diagnosis was 34 years (range 27–45 years). Of the 26 patients, 73% was diagnosed during pregnancy; one patient (4%) was diagnosed in the first trimester, ten patients (39%) in the second trimester and eight patients (31%) in the third trimester. Seven patients (27%) were diagnosed after delivery, but experienced cancer related symptoms during pregnancy. Median survival after diagnosis was 101 days with a range of 3–352 days. Stage IV disease was present at diagnosis in 90% of the patients; the other 10% had stage III disease. Four patients had recurrent disease during pregnancy and were diagnosed with advanced stage disease at recurrence while pregnant; one melanoma and three astrocytomas.

**Table 1:** Patient characteristics

Patient	Tumour	Parity	GA a.d. (wks)	Stage/grade a.d. (TNM/FIGO)	Survival after diagnosis (days)	One-year survival in general (%)	Patient/doctor delay in diagnosis	Patient/doctor delay in treatment	Obstetrical outcome
1	Adrenal carcinoma	G5P3	Pp	IV	133	Unknown	Inconclusive	No	GA 36 weeks; induction for suspicion of HELLP syndrome, but were metastasis.
2	Astrocytoma	G1P0	Pp	II	92	84	Yes, both	Yes, doctor	GA 38 weeks; induction for HELLP syndrome
3	Breast cancer	G2P1	Pp	IV	9	72	Yes, doctor	No	GA 37 weeks; SVD
4	Breast cancer	G9P6	12	IV	310	72	NA	No	GA 32 weeks; emergency CS when patient was found in coma.
5	Breast cancer	G3P2	Pp	IV	126	72	Yes, patient	Yes, patient	GA 39 weeks; induction for excessive skeletal pains
6	Cervical carcinoma	G2P1	25	IV	352	44–54	No	No	GA 34 weeks; maternal deterioration
7	Cholangio- carcinoma	G3P0	28	IV	81	13	Yes, doctor	No	GA 28 weeks; CS for fetal distress
8	Esophagus carcinoma	G5P4	28	IV	78	20	No	No	GA 36 weeks; induction for therapy planning
9	Esophagus carcinoma	G1P0	17	Unknown	110	29	NA	Yes, doctor	IUFD at GA 22 weeks
10	Gastric carcinoma	G3P1	23	IV	78	17	No	No	GA 29 weeks; CS for maternal deterioration and breech position
11	Gastric carcinoma	G2P1	18	IV	171	17	Yes, doctor	No	GA 18 weeks; immature delivery after surgery.
12	Glioblastoma multiforme	G14P10	16	IV	87	38	No	No	IUFD at GA 27 weeks
13	Glioblastoma multiforme	G2P1	28	IV	270	38	Yes, doctor	Yes, patient	GA 34 weeks; CS for twin and maternal deterioration
14	Glioblastoma multiforme	G4P3	16	IV	15	38	Yes, doctor	No	Died while pregnant (GA 18 weeks)
15	Lung carcinoma	G2P1	36	IV	149	22	Yes, both	NA	GA 36 weeks; induction for therapy planning
16	Melanoma	G1P0	26	IV	15	32	No	NA	Died while pregnant (GA 29 weeks)
17	Melanoma	G2P1	21	IIIC	154	82	No	Yes, doctor	Term; SVD
18	Melanoma	G4P2	Pp	IV	47	32	Yes, doctor	No	GA 40 weeks; SVD
19	Melanoma	G1P0	16	IV	167	32	No	Yes, patient	GA 32 weeks; fetal distress
20	Non-Hodgkin lymphoma	G3P2	Pp	IVB	20	67	Yes, doctor	No	Term; SVD
21	Ovarian carcinoma	G2P1	34	IIIB	176	77	Yes, patient	No	GA 35 weeks; induction for therapy planning
22	Pancreatic carcinoma	G1P0	24	IV	37	10	No	No	GA 28 weeks; SVD
23	Pilocytic astrocytoma	G2P1	17	I	3	89	No	No	Died during pregnancy (GA 17 weeks)
24	Rectum carcinoma	G2P1	37	IV	134	46–70	Yes, doctor	No	GA 37 weeks; induction after diagnosis.
25	Satellite tumour CNS	G1P0	21	III	130	61	No	Yes, doctor	GA 34 weeks; CS for maternal deterioration
26	Unknown primary tumour	G1P0	Pp	IV	82	–	Yes, NA	No	GA 26 weeks; CS for HELLP. Neonatal death after 4 days.

*Pp* postpartum, *G* gravidity, *P* parity, *a.d.* at diagnosis, *NA* not assessable, *GA* gestational age, *HELLP* hemolysis, elevated liver enzymes, and low platelet count, *CS* caesarean section, *SVD* spontaneous vaginal delivery, *IUFD* intra-uterine fetal death, *CNS* central nervous system

Standard curative therapy was applied in nine cases. Four patients received adjusted treatment because of pregnancy or complications. In six cases, the patient decided to postpone treatment until a higher gestational age (GA). Four of these patients were still induced preterm. Palliative care was the only option for six patients. For one patient, data on therapeutic decision-making was not available.

### Obstetrical outcome

**Fig. 1:**
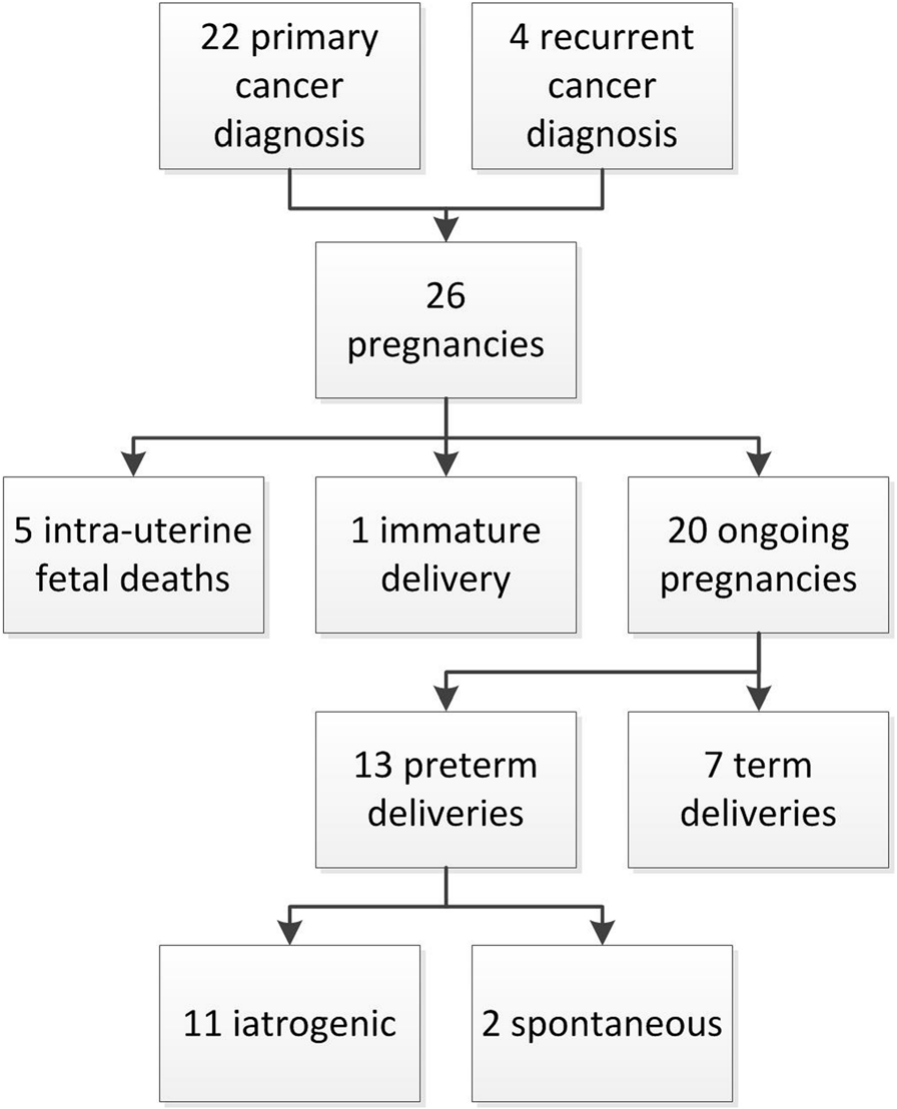
Obstetrical outcome for all 26 pregnancies

Obstetrical outcome was available for all 26 patients with detailed case information (Fig. 1). Five pregnancies were complicated by intra-uterine fetal death: 1) at a GA of 17 weeks, when mother died of an astrocytoma, 2) at a GA of 18 weeks, when mother died of a glioblastoma multiforme, 3) at a GA of 22 weeks after suboptimal intubation during surgery, 4) at a GA of 27 weeks, when mother was admitted with a status epilepticus due to progressive glioblastoma multiforme, and 5) at a GA of 29 weeks, when the mother died suddenly at home due to metastatic melanoma. One patient experienced an immature delivery at a GA of 18 weeks after a bilateral laparotomic oophorectomy.

Of the 20 ongoing pregnancies ending in a live birth, one was a twin pregnancy resulting in 21 live new-borns. However, one of the children died 4 days after caesarean section at a GA of 26 weeks due to complications of extreme preterm delivery. Caesarean section was performed because of presumed HELLP syndrome. After delivery, the complaints progressed and stage IV cancer of unknown origin with liver metastases was diagnosed.

Median GA at delivery of all 20 ongoing pregnancies was 35 weeks (range 26–40 weeks). Thirteen (65%) of these ended preterm, of which 11 deliveries (85%) were vaginally induced (*n* = 3) or terminated by caesarean section (*n* = 8). Reasons for iatrogenic preterm delivery were maternal deterioration (*n* = 4), therapy planning (*n* = 4) or obstetrical complications (*n* = 3).

### Doctor-related and patient-related delay

Ten medical specialists dealing with pregnant cancer patients from six different medical centres independently scored all 26 case descriptions on delay in diagnosis and treatment. The score was considered conclusive if six or more specialists shared the same opinion.

See Table 2 for an overview of the types of delay. In 65% of patients (*n* = 17) delay was found to be present, 50% (*n* = 13) or all patients were found to have delay in diagnosis and 27% (*n* = 7) in starting therapy. Three patients were considered to have both types of delay. Regarding the other nine cases, six showed no signs of delay, two had not enough information available for adequate evaluation and one score was inconclusive. Even in patients with recurrent disease, delay in diagnosis occurred in 50%. In one of these patients with recurrent astrocytoma, delay was caused by doctors in a non-academic centre where symptoms like increasing blood pressure, headache and general seizure were interpreted as eclampsia, for which delivery was induced. In the other patients with delay in diagnosis with recurrent melanoma, progressive back pain and pain on her ribcage at a GA of 35 weeks, was treated with pain medication until 2 weeks after delivery. Diagnostic investigations showed wide spread melanoma metastasis including bones and lungs.

**Table 2:** Delay in diagnosis and treatment

Type of delay	No.	%
Overall delay	17	65
Delay in diagnosis	13	50
- Doctor-related^a^	8	62
- Patient-related^a^	2	15
- Doctor- and patient related^a^	2	15
- Not assessable^a^	1	4
Delay in therapy	7	27
- Doctor-related^a^	4	15
- Patient-related^a^	3	12

^a^Of all patients with delay

Inter-observer agreement on the presence of delay in diagnosis was found to be substantial, with a Fleiss Kappa score of 0.71. The agreement for the delay in therapy was less, with a score of 0.33, which is a fair agreement.

In the cases where delay in diagnosis was caused, the origin was evaluated to assess whether the delay was caused by the involved health care provider or by the patient. In eight of 13 patients with delay in diagnosis, doctor-related delay was considered to have influenced the time between presentation of symptoms and diagnosis. In two additional cases, both the health care provider and the patient caused delay. For the ten patients with doctor-related delay, delay was caused by the midwife (*n* = 5), general practitioner (*n* = 2) and by doctors from a non-academic centres (*n* = 2) and in two patients, multiple non-academic health care workers caused delay. In nine cases, symptoms were described as pregnancy related complaints, and in one patient, a wrong diagnosis was made based on additional diagnostic tests. In one patient, delay was caused by a long period between referral to a hospital and actual diagnostic evaluation. Patient-related delay in diagnosis was present in only two cases, and in one case enough information was not available to evaluate the cause of delay.

Doctor-related delay in therapy was found in four of the seven cases. In the other three cases, patients refused the advised treatment during pregnancy and wished to delay until after delivery.

## Discussion

This is, to our knowledge, the second study to evaluate specific cancer related MMR and the first to report on delay in either diagnosis or treatment and obstetrical outcome in this specific population. The cancer related MMR in the Netherlands between 2001 and 2012 was 1.23 per 100,000 live births, with the highest incidence of cause related death due to brain tumours, gastro-intestinal tumours and melanomas. Delay in diagnosis was more frequent than delayed treatment, and was mainly caused by health care providers. Of the 77% ongoing pregnancies, 65% delivered preterm, often after induction of labour for oncological reasons. One neonatal death occurred.

The only other article published on cancer related maternal mortality, in 1990 by Sachs et al. [12], reported on their population-based study in the USA between 1954 and 1985 and found a cancer related MMR of 1.44. They defined maternal mortality as death during pregnancy or within 90 days after delivery. Because this is different from the current WHO definition used in our study, comparing these results to this present study is not feasible [12].

The outcome for pregnant patients with cancer is not different from non-pregnant patients when corrected for stage and age at diagnosis [7]. Previous literature has reported that patients with cancer during pregnancy present with a more advanced stage of disease due to delay in diagnosis [5, 6]. Since stage of disease at diagnosis is strongly correlated to prognosis, this delay may contribute to a worse maternal outcome and should be avoided where possible. For some tumours, the effect of delay on the prognosis is less, since lower stage of disease still has a poor prognosis. However, even in these cases, prognosis is better when diagnosed in an earlier stage.

Delay in therapy until after delivery may postpone adequate therapy for the mother, thereby contributing to a worse maternal prognosis. If preterm induction of labour is aimed for to start therapy postpartum, the prognosis of the unborn child is influenced as well. A recent study [8] showed that there was no difference between neuropsychological development between children exposed to chemotherapy in utero and healthy controls. In fact, preterm birth had a bigger impact on neuropsychological outcome in both groups with an increase of 2.6 IQ points per week gestation at birth [8]. In our study population, delay in diagnosis may have contributed to a more advanced stage of disease requiring systemic therapy in order to improve survival. In 27% of the cases, therapy was delayed until spontaneous delivery or until a certain GA was reached and pregnancy could be terminated. Raising awareness on cancer in pregnancy and the possibilities in diagnostic and therapeutic treatment modalities among health care providers may help in reducing the morbidity and mortality of these patients and their children.

One of the main limitations of our study include that reporting fatalities to the Dutch MMC is on a voluntary basis. Even though the committee is well known among gynaecologists, it is possible that a year after pregnancy, the relation between death and pregnancy by other medical professionals may be overlooked. Cross checking with the CBS database revealed only two more cases, but the actual number of cancer related (late) mortalities might be higher than reported. Furthermore, there are some difficulties in studying delay, as the extent of delay for one tumour will not have the same consequences as delay for another, more aggressive, tumour and if curative therapeutic options are not available, delay will not change the fatal outcome. However, the various medical specialists reached an overall agreement on the occurrence of delay, which makes our findings more reproducible. Our study population is small, because of the low incidence of cancer related maternal mortality.

We cannot exclude that treatment in a multidisciplinary team may influenced outcome. Practitioners with experience in oncologic treatment in pregnancy will probably hesitate less to start treatment, but they are often dependent on referral from first-line healthcare workers and they cannot change the stage of cancer. This emphasizes that awareness in all practitioners is essential.

## Conclusions

In conclusion, cancer related maternal mortality is rare but contributes to high neonatal morbidity and mortality rates, mainly due to iatrogenic preterm delivery. Delay in diagnosis and treatment occurs frequently in this group and is mainly caused by health care providers. This can only be resolved by increased awareness among health-care professionals.

## Abbreviations

CBS: Central Bureau of Statistics
GA: Gestational age
MMC: Maternal Mortality Committee
MMR: Maternal mortality rate
